# Supplementary figures and images for: An acute microglial metabolic response controls metabolism and improves memory
Source: eLife. 2024 Dec 3;12:RP87120. doi: 10.7554/eLife.87120 (PMC11614388; doi:10.7554/eLife.87120)

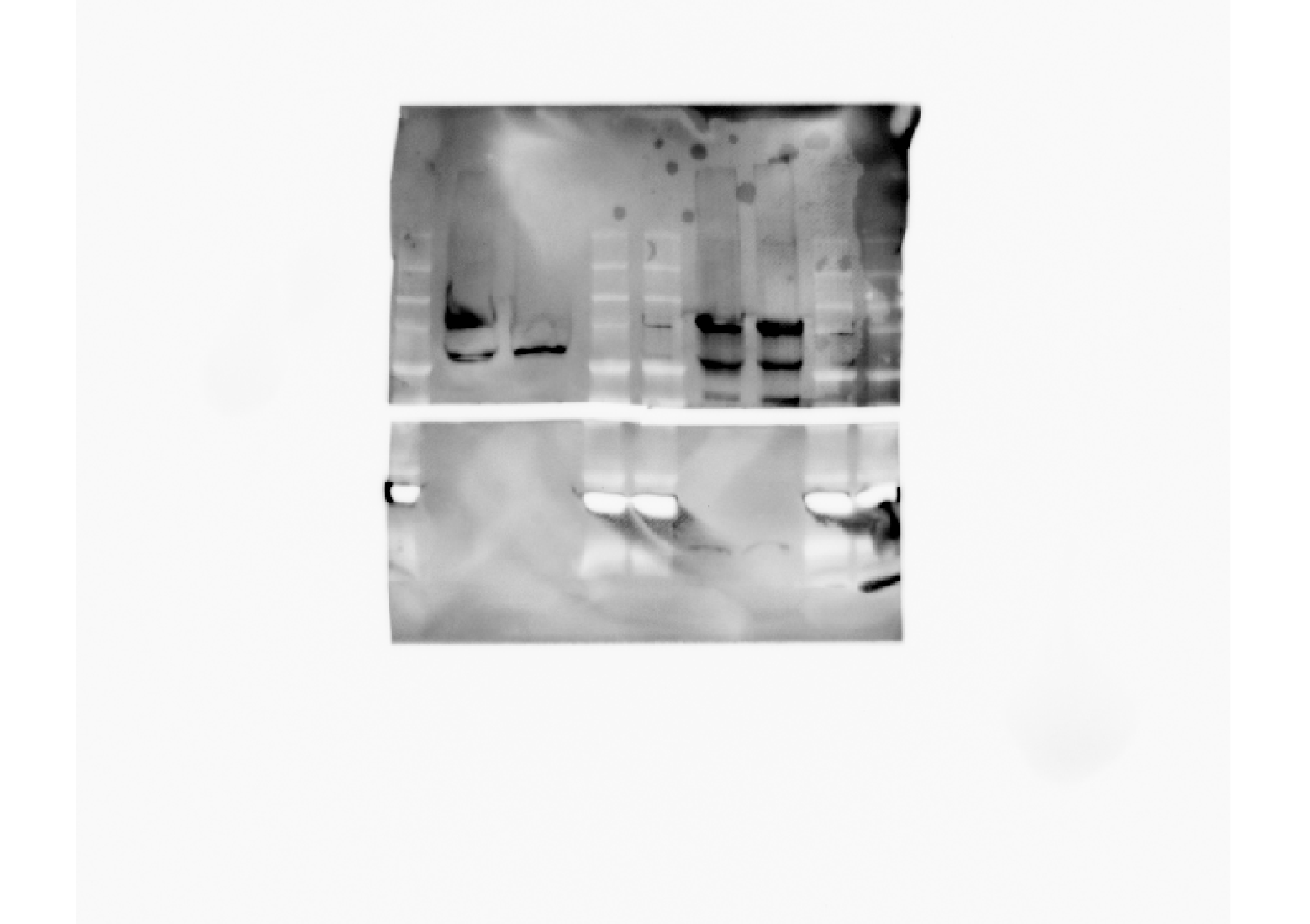

Supplement: Figure 3—source data 1. [file elife-87120-fig3-data1.jpg]

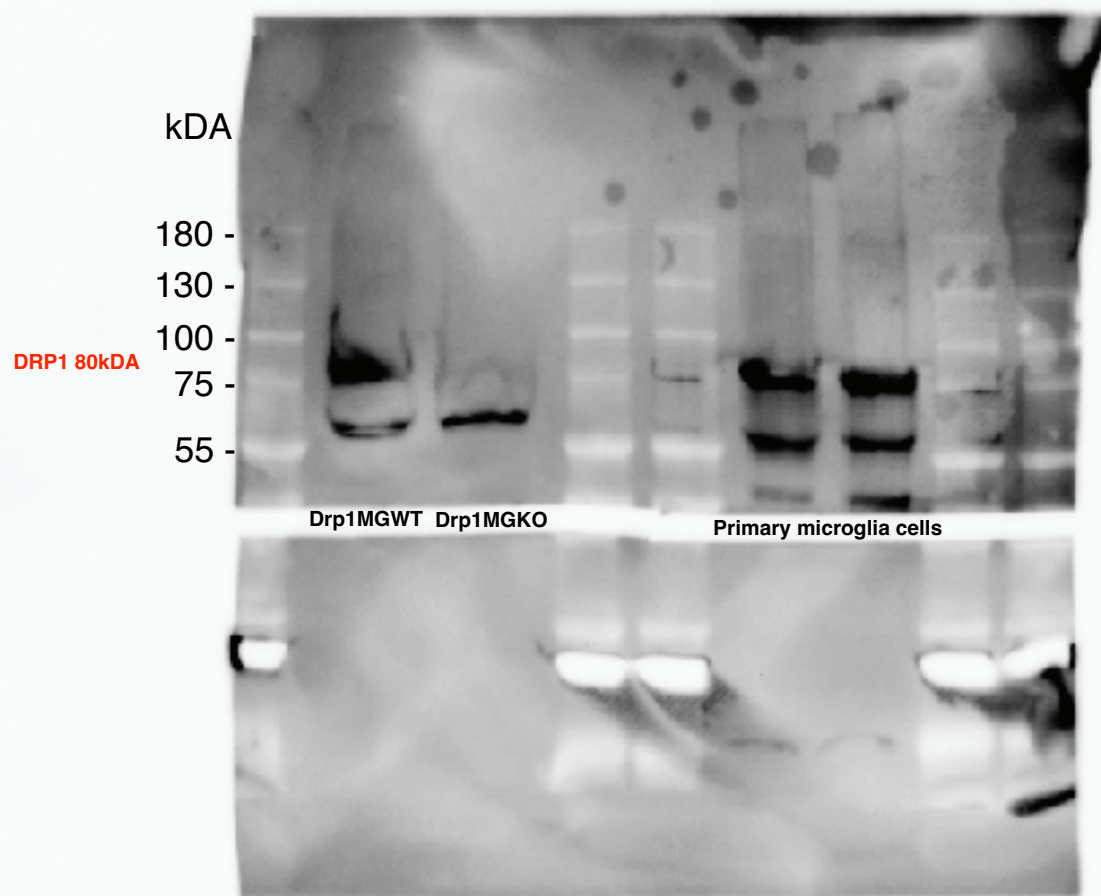

Supplement: Figure 3—source data 2. [file elife-87120-fig3-data2.pdf]
